# Injectable anti-malarials revisited: discovery and development of new agents to protect against malaria

**DOI:** 10.1186/s12936-018-2549-1

**Published:** 2018-11-01

**Authors:** Fiona Macintyre, Hanu Ramachandruni, Jeremy N. Burrows, René Holm, Anna Thomas, Jörg J. Möhrle, Stephan Duparc, Rob Hooft van Huijsduijnen, Brian Greenwood, Winston E. Gutteridge, Timothy N. C. Wells, Wiweka Kaszubska

**Affiliations:** 1 Medicines for Malaria Venture, Route de Pré Bois 20, 1215 Geneva, Switzerland; 2 Drug Product Development, Janssen R&D, Johnson & Johnson, Turnhoutseweg 30, 2340 Beerse, Belgium; 3 Department of Science and Environment, Roskilde University, 4000 Roskilde, Denmark; 4 Faculty of Infectious and Tropical Diseases, London School of Hygiene and Tropical Medicine, London, UK

**Keywords:** Malaria, *Plasmodium*, Chemoprotection, Prophylaxis, Liver schizont, Intra-muscular, Target candidate profile, Target product profile

## Abstract

Over the last 15 years, the majority of malaria drug discovery and development efforts have focused on new molecules and regimens to treat patients with uncomplicated or severe disease. In addition, a number of new molecular scaffolds have been discovered which block the replication of the parasite in the liver, offering the possibility of new tools for oral prophylaxis or chemoprotection, potentially with once-weekly dosing. However, an intervention which requires less frequent administration than this would be a key tool for the control and elimination of malaria. Recent progress in HIV drug discovery has shown that small molecules can be formulated for injections as native molecules or pro-drugs which provide protection for at least 2 months. Advances in antibody engineering offer an alternative approach whereby a single injection could potentially provide protection for several months. Building on earlier profiles for uncomplicated and severe malaria, a target product profile is proposed here for an injectable medicine providing long-term protection from this disease. As with all of such profiles, factors such as efficacy, cost, safety and tolerability are key, but with the changing disease landscape in Africa, new clinical and regulatory approaches are required to develop prophylactic/chemoprotective medicines. An overall framework for these approaches is suggested here.

## Introduction

One of the challenges of malaria control is to be able to provide protection for vulnerable populations. In recent years, there has been great progress in the use of drug regimens to prevent infections in children. However, these require frequent drug administration. One alternative that has been tested is the use of long-acting injectable formulations. Although there are currently no long-acting injectable medicines in development for malaria, new formulation technologies, similar to those developed for prophylaxis against HIV, might point the way to new approaches against this disease. In addition, recent developments in monoclonal antibody technology may be applicable to protect against malaria, especially in vulnerable populations. This paper discusses how such treatments would fit the target product profiles for malaria, and a regulatory pathway for their development. Since they pose similar challenges and possibilities from this perspective, the discussion of small molecules and antibodies was combined.

## Background

Over the last decade, there has been a considerable increase in the portfolio of new molecules which are being developed for the treatment of malaria [1, 2]. New paradigms of screening [3–8] have led to another generation of molecules progressing to clinical development, aimed at producing new medicines which overcome the current problems of multi-drug resistant malaria [9–11] and which also simplify therapy from the current 3-day therapy, to single exposure cures. Protecting vulnerable populations from clinically significant infections is also a key aspect of malaria control and elimination [12]. Although a highly effective vaccine is the ultimate goal of much basic malaria research, the absence of a sterilizing immune response to naturally acquired disease shows how difficult such a target is likely to be [13]; current candidate vaccines provide a protective efficacy in the range of 30–50% [14].

Malaria chemoprophylaxis can be achieved by a variety of different mechanisms. Causal prophylactics target the asexual hepatic stages of malaria. Atovaquone (a *Plasmodium* mitochondrial bc_1_ inhibitor binding to the Q_0_ site of the complex), pyrimethamine and cycloguanil and its prodrug proguanil (selective inhibitors of dihydrofolate reductase, DHFR), sulfadoxine (an inhibitor of *Plasmodium* dihydropteroate synthase, DHPS) and the 8-aminoquinolines primaquine and tafenoquine are the only causal prophylactics with proven clinical efficacy. In suppressive chemoprophylaxis, the parasite is killed once it enters the erythrocytic stages of the lifecycle, stopping the infection at a low level of parasitaemia before it becomes clinically significant. Inhibitors of beta-haematin formation, such as chloroquine and mefloquine, and (plastid) ribosomal inhibitors, such as doxycycline, are suppressive chemoprophylactics, and must be given for at least 2 weeks after leaving a malaria-endemic area to clear any parasites that emerge from the liver after a person has left the endemic area. Currently, the predominant oral prophylactics used by travelers are (rarely) mefloquine, doxycycline and atovaquone–proguanil. These were initially developed as treatments for uncomplicated malaria [15, 16]. They were subsequently shown to have good protective efficacy when delivered at lower doses on a weekly basis, in the case of mefloquine, and a daily basis in the case of atovaquone–proguanil, respectively [17, 18].

Protective vaccine strategies have focused on developing an immune response which blocks the initial infection, using the parasite antigen CSP-1 (circumsporozoite protein; [19]). Although a high level of protection has not currently been achieved with such vaccine candidate approaches, they have provided a proof of concept that antibodies can be generated that prevent the initial infection of the hepatocyte by sporozoites [20–23]. Efficacious vaccines would hold tremendous promise for prophylaxis, but unlike many viral infections, malaria is not a disease where natural infection results in sterilizing immunity, indicating that the bar to the identification of a highly effective vaccination regimen for malaria remains very high.

Identifying new classes of anti-malarial chemoprotective molecules targeting either blood- or liver-stage has historically been limited by the lack of known molecular targets and of high-throughput screening methods. However, in the last 10 years there has been a dramatic increase in the deployment of high density, phenotypic, cell-based high-throughput screens of *Plasmodium* blood stages [24] and new chemotypes, which are active against the blood stages of *Plasmodium* infection, have been identified [25]. These families of molecules can then be tested for activity against other stages of the parasite life cycle including hepatic schizonts. Examples in the Medicines for Malaria Venture (MMV) pipeline of compounds having both blood and liver stage activity [1] include: the DHODH (dihydroorotate dehydrogenase) inhibitor DSM265 [26, 27], the PI-4 kinase inhibitor MMV048 (MMV390048; [28]) and the EF2 (Elongation Factor 2) inhibitor DDD498 (DDD107498; [29]), now also known as M5717. These molecules have helped identify new molecular targets in liver schizonts. Other scaffolds such as KAF156 [30] have good activity against hepatic schizonts. However, although the main mechanism of resistance generation involves PfCARL, KAF156’s molecular site of action is still to be elucidated [31]. More recently, high-throughput screens of the hepatic schizont stages have been carried out for murine parasites [32], increasing the possibility of the identification of compounds which are selectively active against hepatic schizonts. The immediate challenge is to develop high-throughput hepatic schizont stage assays using sporozoites from *Plasmodium falciparum*, and progress in this direction is being made. This is particularly important in order to confirm activity of compounds without asexual blood stage activity, and to avoid a focus on murine-malaria specific prophylaxis.

Work on injectable depot anti-malarials was a key part of the previous malaria eradication campaign with the development of 4-4′-diacetylaminosulphone (DADDS), a long-acting prodrug of the DHPS inhibitor dapsone, and cycloguanil pamoate (CI-501; [33, 34]). Clinical protection over 3–5 months was achieved in the published clinical trial, but the dose had to be delivered deep into the gluteal muscle tissues (350 mg in adults) and caused local discomfort and abscesses [35]. Failure to protect was assumed to be due to cross resistance with pyrimethamine, which was emerging at the time. Interest in long-lasting anti-malarials was renewed in 1976, supported by the UNDP/World Bank/WHO special program for Research and Training in Tropical Disease [WHO CHEMAL/SC(33)77.3 item 1.2] and the US Army Medical Research and Development Command. These groups continued to focus on blood schizonticides for injection, but no molecules were brought forward for testing in field studies. Tafenoquine/WR 238605, an 8-aminoquinoline originally discovered by The Walter Reed Army Institute for Medical Research has recently been approved by the US FDA (Food and Drug Administration) as a weekly oral prophylactic against *Plasmodium vivax* and *P. falciparum* (Arakoda^®^, 60 Degrees Pharmaceuticals) and for a single-dose radical cure indication (i.e. the treatment of the liver stage, preventing *P. vivax* relapse), as Krintafel^®^, by GSK. Both uses require a test for a patient’s G6PD (glucose-6-phosphate dehydrogenase) deficiency status as a safety measure. No further clinical work on injectables has been reported. In the 1980s, Chinese scientists investigated the use of pyronaridine given by injection, with a total intramuscular dose of 300–400 mg being given in two or three injections. The drug was rapidly absorbed and provided an efficacious concentration that remained for an extended period given the elimination half-life of 60 h [36].

More recently, there has been a significant effort to develop anti-virals that provide protection against HIV infection in high risk individuals using daily oral dosing, termed pre-exposure prophylaxis, PrEP [37]. However, clinical trials have shown variable rates of efficacy with low rates of protection, correlated with non-adherence to the daily oral drug regimen [38]. Long-acting injectables have been developed based on the reverse transcriptase inhibitor cabotegravir (GSK1265744) and the integrase inhibitor rilpivirine. These provide an opportunity for better adherence through protection when administered on a monthly or even less frequent basis [39]. Similar issues have been addressed in the realm of anti-psychotic medicines, where non-adherence to daily oral regimens is also a challenge [39].

When considering new molecules that could be used in chemoprotection, it is important to consider their ultimate use, since this drives the regulatory strategy. As mentioned earlier, previous generations of medicines, such as mefloquine and atovaquone–proguanil, were first approved for case management, and were only subsequently approved for use at lower doses for prophylaxis. However, if the priority is to deliver new medicines for prophylaxis rapidly, one alternative approach would be to pursue a path towards initial registration directly for prophylaxis; this would require a new clinical and regulatory approach. The key question is to define how large and diverse a population will need to be exposed to a new medicine before the risk–benefit balance is established and considered adequate, and this ratio is clearly different between a medicine used for treatment and one for prophylaxis. Another question relevant to development is how to protect the new drug; any deployment strategy must also take into consideration the need to protect the drug against the emergence of resistance [40]. The assumption made here is that a combination of two molecules with different mode of action would be ideal to protect against the selection of resistance. It is conceivable to use a single molecule, if the case could be made that the potential development and transmission of resistance in human subjects would be minimal [41]. Such a case would be strengthened by targeting non-replicating or low-copy number stages, such as the sporozoite. The key factor in reducing the risk of resistance generation is avoiding exposure of the drug in subjects with existing parasitaemia. At first glance the ideal medicine would prevent development of the blood-stage of the infection, by killing all parasites before they escaped from the liver. However, a subclinical blood-stage infection may also drive some protective immunity, and so could even be advantageous, as long as the prophylactic drug achieved clearance of the blood stage infection.

Previously, targets have been proposed for the characteristics of molecules which could be used as oral medicines in prophylaxis, and a target candidate profile for molecules with hepatic stage activity (TCP-4); [42]). It seemed timely, therefore, to revisit the characteristics of an injectable therapy for prophylaxis for a number of reasons. First, over the last 2 years there has been an increasing interest in the potential for long-acting injectables, perhaps driven by the success in the antiretroviral arena, and also the re-assessment of the difficulty of obtaining a vaccine with high efficacy and a long duration of protection. Second, there has been a renewed interest in the use of monoclonal antibodies to protect against the establishment of a liver stage infection. This is largely driven by the availability of monoclonal antibodies against the Circumsporozoite Antigen-1 (CSP-1), coming from the recent RTS,S clinical trials. Third, there has been dramatic progress in protecting children from infection in the Sahel by Seasonal Malaria Chemoprevention (SMC) since the new strategy was recommended by the WHO in 2012 [43]. Here, administration of full treatment courses of 3 days of amodiaquine and one dose of sulfadoxine–pyrimethamine were delivered to 15 million children in 12 countries in 2016 [44]. This raises the question as to whether such chemo*prevention* could be replaced by a more easily delivered chemo*protection* regimen (chemoprevention is use of a full treatment course for prophylaxis, chemoprotection is using a specifically-designed and tested prophylaxis regimen). A once-per-season injectable regimen would certainly be worth considering. Moving to chemoprotection (prophylaxis), rather than simply using a full treatment course monthly, requires a new regulatory and clinical approach, and early discussions on this strategy have taken place with regulators during the past 2 years.

In a discussion of potential new medicines there are always many moving parts. A common framework for discussion and agreement on the ideal and minimally acceptable protective efficacy and other qualities of new medicines is important, given the lengthy development required from discovery to launch. In the world of injectable chemoprophylaxis, such a discussion is especially important, given the overlap between the potential uses for injectable small molecules, therapeutic antibodies, and vaccines. Based on these insights, the Target Candidate and Product Profiles (TPPs) presented here will need to be refined and updated in discussions within the malaria community, as and when new data become available.

## The different modalities for deploying malaria prophylaxis

There are multiple uses for an injectable anti-malarial prophylaxis (Table 1). The first is to protect populations such as migrant workers, soldiers, tourists and university or boarding school students originating from a malaria-free area from becoming infected when they travel to areas with endemic malaria. Historically prophylaxis has been considered as a premium priced market, largely targeting western tourists and the military, who can afford to pay $5/day for protection. However, as the impact of malaria elimination has progressed, there are now many areas, especially in Africa, where it is relatively easy to travel from a low endemic to a high endemic region, such as from Southern to Northern Zambia. This latter group is an important and expanding group in the malaria agenda. With the progress of the elimination agenda there will be a need for affordably-priced protection for Africans who move from low to high transmission areas. Another important group is residents of non-endemic areas, such as Europe or the USA, whose families come from an endemic area and who return to visit their families in a malaria endemic area. This group is responsible for the highest proportion of cases of imported malaria in Europe.

**Table 1:** Summary of potential uses for injectable prophylactic medicines

Use case	Description	Target population	Comments
(1) Travellers	Residents of regions of very low or no malaria incidence travelling to malaria endemic areas	Initially, all adults and children > 5 years	Increasing numbers of travellers within Africa, given increased GDP; increasing numbers of Africans in areas of low transmission
(2a) Malaria epidemic	Re-emergence of malaria in zones which had been previously declared malaria free	Entire population	Need demonstration of safety in first trimester of pregnancy; deployment during ‘maintaining zero’
(2b) Febrile epidemic	Protection of a population from malaria during epidemics such as Ebola	Entire population	Need demonstration of safety in first trimester of pregnancy. Currently maintained as monthly ACT. Value only if injections offer longer protection
(3) Protection from infection of subjects in high transmission zones	Replacing the SMC regimens of monthly protection by SP-AQ given currently to children under 5 in the Sahel with true chemoprotection	Children < 10 years	No requirement for safety demonstration in first trimester of pregnancy; combination would ideally have causal prophylaxis (preventing blood-stage and liver stage activity)

The second use is to protect a non-immune population within their area of residence, who are suddenly exposed to a malaria outbreak or epidemic. This is one of the major concerns late in any eradication effort, in places where ‘maintaining zero’ is the priority [45]. In a situation where areas are malaria-free, but the mosquito population is still abundant, there may be a need to rapidly protect populations. In such situations both adults and children will need to be protected. It may not be possible to test the population for pregnancy status, so the ultimate challenge here is to have a medicine which has a high probability of being safe in the first trimester of pregnancy. A variant on this is the protection of populations from malaria during a pandemic outbreak of febrile disease, such as Ebola. During the 2013–2016 outbreak of Ebola in West Africa, many patients who presented with malarial fevers were triaged into the Ebola facilities, where they became infected with Ebola [46–48], resulting in patients not seeking malaria treatment and an increase in deaths due to malaria [49]. Monthly presumptive treatment with artemisinin-based combination therapy (ACT) was used in at-risk populations in Liberia and Sierra Leone. An injectable prophylaxis could be useful in this kind of situation, if it gave a longer period of protection than oral medication.

The third potential use for an anti-malarial chemoprotective agent is to protect vulnerable populations in areas of high malaria incidence, and this is somewhat distinct from the other uses. Over recent years, there has been a massive deployment of sulfadoxine–pyrimethamine plus amodiaquine (SP–AQ) for SMC. Chemoprevention is defined as giving a full curative course of treatment. This is given over 3 days, to each child under 5 years old, monthly throughout the rainy season in the Sahel (to 13 million children in 2016), to reduce the incidence of symptomatic malaria in this vulnerable population. Since a large part of the cost of deployment of SMC is the delivery, a better treatment would be a once-monthly, single oral medication. A longer-acting injectable would have to offer significant advantage, which is why the ideal threshold for this use has been set at 3 months in the target product profile shown in Table 2. The nuance here is that the clinical development strategy of the injectable would be for protection, as discussed below, rather than using a standard treatment dose and, therefore, in theory the dose would be better titrated to the needs of protection.

**Table 2:** TPP for an injectable prophylactic medicine for malaria

Parameter to be clinically evaluated for the combination	Minimum essential	Ideal
Antimalarial effects	Blood schizonticides with at least one molecule also having causal prophylactic activity (killing hepatic schizonts)	Both molecules should have causal prophylactic, blood schizonticidal and transmission-blocking activities
Mechanism of action	Two partner drugs without cross resistance	Two partner drugs have different modes of action, so no cross resistance
Dosing regimen	Once per month, intramuscular, with an acceptable injection volume	Once per 3 months, intramuscular or sub-cutaneous with an acceptable injection volume
Rate of onset of action	Protection, within 72 h of initial injection	Immediate protection (no lag prior to onset of action)
Clinical efficacy	≥ 80% protective efficacy	≥ 95% protective efficacy: reduction in incidence of symptomatic malaria No drug-resistant parasites identified in volunteer infection studies still capable of transmission
Drug–drug interactions	No unmanageable risk in terms of solid state or PK interactions	No risks in terms of solid state or PK interactions with other co-administered PrEP or therapeutics
Safety and tolerability	No drug-related SAEs; minimal drug-related AEs—i.e., not resulting in clinical study exclusion. No unacceptable pain, irritability of inflammation at injection site, especially injection abscesses	Idem
Use in patients with reduced G6PD activity	Testing not required as no enhanced risk in mild-moderate G6PD deficiency	Testing not required as drugs not linked to haemolytic risk
Use in infants/children	Use in children > 6 months old	Use in infants, children and adults
Formulations	Suitable for intramuscular injection with minimal preparation; maximum volume of 2 mL for adults and 0.5 mL for infants, administered with 27 gauge needle; partner drugs can be injected separately	Liquid pre-filled injection device for intramuscular; maximum volume of 1 mL for adults and < 0.5 mL infants administered with 27–30 gauge needle; fixed dose combination of the drugs; or subcutaneous injection if volumes smaller than above for intramuscular injection
Cost of treatment	< 5 USD per injection	≤ USD 1 for infants, USD 2 for children, USD 4 for adults
Shelf life of formulated product (ICH guidelines for zones/IVb)	≥ 2 years	≥ 3 years

*PK* pharmacokinetic, *(S)AE* (severe) adverse event, *ICH* International Conference on Harmonization

The regulatory strategy in each case would be to file for an indication of ‘Prophylaxis against *Plasmodium falciparum* malaria’. This would be defined as prevention of malaria infection in subjects travelling from geographical areas with no, or very low risk of malaria infection, to geographical areas with significant risk of malaria infection. The clinical development strategy supports the filing by collecting data to demonstrating acceptable efficacy, safety and tolerability in the target populations.

## Defining a product profile for injectable prophylaxis

The discussion of a TPP for an injectable prophylactic medicine is informed by the uses described above. The final acceptance of an injectable product is guided by experience with two other therapeutic or preventative approaches. First, site injection tolerability, in terms of needle size, and injection volume should be driven by a favourable comparison to vaccines. The most advanced malaria vaccine, Mosquirix^®^ (RTS,S–AS202) has been approved by the European Commission after having been given a positive scientific opinion by the CHMP (Committee for Medicinal Products for Human Use at the EMA (European Medicines Agency), and is about to undergo Phase IV in-country studies. The current course of vaccination involves four 0.5 mL intramuscular injections with a 25-gauge needle, at a cost of around $5 per injection in infants. Second, in HIV infections, where an effective vaccine has also been a major challenge, pre-exposure prophylaxis, or PrEP, using an injectable is being studied [37, 50]. Although large injection volumes and needle have been found acceptable, for example, in the injection of penicillin-G benzoate crystals for bacterial infections, such as mass treatment against yaws [51], a new medicine for children protecting against malaria would need to be more child-friendly to be acceptable for repeat use. Intravenous injection of the PfSPZ vaccine [52] with a very fine needle has been very well tolerated even in very small children, but i.v. injection would probably not be the ideal route to achieve a prolonged duration of protection. From a formulation and pharmaceutical perspective, intramuscular or subcutaneous depot approaches are preferred to achieve the desired long duration with new chemical entities.

The level of efficacy required for a new product can be gauged by the efficacy of current prophylaxis. In comparative, randomized clinical trials, nonimmune adults, adolescents and children (≥ 11 kg) visiting malaria-endemic regions and receiving once-daily atovaquone/proguanil (250/100 mg in adults and dosage based on body weight in children < 40 kg), had no cases of falciparum malaria for 28 days [53], comparable efficacy was also seen in other studies [54, 55]. Doxycycline provides 84–99% protection (studies cited in [41]). Lessons from the vaccine community also suggest that a protective efficacy of ≥ 80% for a once per 3-month injection might be acceptable. The current working hypothesis is that a molecule either preventing or treating hepatocyte stage infection would be useful when looking at new chemical entities. However, molecules with pure blood stage activity (chloroquine and mefloquine) have been the mainstay of oral prophylaxis and so whether this would be acceptable for an injectable remains a question for further discussion.

The clinical safety requirements of a long-acting injectable for prophylaxis are much more difficult to define precisely in advance of data. The two overriding principles are that first, the drug will be given to subjects with no overt disease, and so the safety thresholds should in general be similar to those required for vaccines. This will inevitably lead to the need for considerable phase IV activities. The second aspect relates to the long plasma residence time of pharmacologically active material. Appropriate safeguards have to be in place for the care of subjects, should a severe adverse event occur, and clearly such an event would preclude further development of the medicine for populations in low-resource settings. However, it is important to underline the fact that the safety considerations related to an injectable drug driving exposure over several months are very similar to the safety considerations related to an oral drug driving exposure over several months. The ideal medicine is one that can be used to protect all ages; however the development of the medicine for children will require particular care, and the availability of substantial safety data in adults before proceeding to the more vulnerable younger populations.

A target product profile (TPP) including other parameters for an injectable prophylactic medicine for malaria is proposed in Table 2.

## Approaches to finding and developing new small molecule TCP-4 candidates

Compounds with a TCP-4 profile would normally be shown to be highly potent against the hepatic schizonts of *P. falciparum* parasites. The definition of a hit and a lead still follow the criteria discussed previously [56], with compounds showing an IC_50_ of less than 1 µM being considered as potential starting points for medicinal chemistry, aiming for a final potency of less than 10 nM. The key data however are those that increase our understanding of how well this translates into in vivo efficacy, and allowing an early prediction of the human effective dose.

In vivo measurements of efficacy have been performed traditionally using infection with GFP- (green fluorescent protein) or luciferase-expressing *Plasmodium berghei* sporozoites in mice. This assumes equipotent activity between *P. berghei* and *P. falciparum.* A newer model is the FRG huHep chimeric mouse model with engrafted human hepatocytes and erythrocytes. FRG stands for the lack of functional Fah, Rag-2 and Interleukin 2 receptor common gamma chain genes in these animals. This model allows an endpoint based on asexual blood stage infection from the liver [57]. Here, the benchmarking with atovaquone shows that a highly efficacious compound should be able to lower the parasite 18S RNA signal in the liver by 10^7^-fold, or a 200-fold reduction in the bioluminescence signal [58]. DSM265, a novel PfDHODH inhibitor, with demonstrated activity in humans, shows a 10^4^-fold reduction in this model, arguably defining the lower threshold of efficacy. During lead optimization, it is important to be able to assess the human effective dose, i.e. the dose required to produce a similarly effective exposure in humans. In the absence of imaging technologies to determine the time-dependent concentration of a drug within the liver, the definition of the prophylactic concentration has to be based on the plasma concentration, assuming that the plasma to hepatocyte concentration ratio is invariant with plasma exposure. The minimum protective plasma concentration in the mouse model can then be used as the basis for pharmacokinetic modelling of the human exposures. For a pre-clinical orally active compound the ideal threshold is an adult dose of < 100 mg, or < 2 mg/kg. Similar constraints apply to injectables, but driven by a need to be able to deliver the drugs in an acceptable injection volume of < 0.5 mg per drug, as discussed below. Two other biological factors are important. First, the demonstration that clinical selection of mutations is a rare event, or that these mutations are not transmitted. It has been shown that is relatively easy to select for mutations against atovaquone both in vitro and in patients, but that these mutations are difficult to transmit [59]. Second, species selectivity: although 99% of the global burden of disease is currently carried by *P. falciparum*, activity against other species would be an advantage, since many patients have mixed infections that include *P. vivax*, *Plasmodium malariae* and *Plasmodium ovale*.

Selection of a formulation, which may also involve the use of pro-drugs, is critical to enable the maintenance of long-lasting protection. The ideal use case would be a 3-month coverage from a single injection, allowing potential protection for a season in some countries. Thus, compounds having high potency and very low clearance, and with formulations delivering long-term drug release into the systemic circulation are favoured. When considering the product presentation, a number of factors need to be taken into account, including but not limited to: cost of goods, a therapeutic product that can be stored at room temperature in climate zones III and IV, a formulation with a potential for local production, and a formulation that is easy to administer. As such there is no published decision tree that maps the technology path for selecting long-acting injectable formulations. A range of different formulation technologies have been applied in commercial products, such as oil solutions, aqueous micro- and nanosuspensions, in situ forming gels, and micro-particular systems. There is, hence, a good industrial basis to define the most likely options that would fulfil both the technological elements as well as the cost of the product. In resource-limited settings, the first line of formulation will most likely be an oil solution or suspension of the micro- or nano-particulate drug, due to the simplicity of development and production of the formulation, low cost of excipients and potential for thermal sterilization, which would allow local production. The use of an oil-based solution or suspension requires clinically acceptable oils, such as fractionated coconut, castor, corn, cottonseed or olive oils. A key consideration here will be local tolerability [60].

The second option for a long-acting injectable for prophylactic treatment of malaria would probably be an aqueous suspension of either micro- or nanoparticles. The advantage of an aqueous suspension is that there is a well-established relationship between particle size and rate of dissolution. This allows much better control of the rate of release of the drug and, therefore, better matching of the final pharmacokinetics, once the human clearance is known. In the case of oil solutions of the drug, the oil is metabolized by macrophages in the muscle over a period of days to weeks, resulting in an amorphous depot of drug in the tissue. Data from preclinical animal models and in humans have demonstrated that appropriate slow release of a drug can be obtained, although the choice of oil is largely empirical at this stage. The technology associated with aqueous suspensions is well established, i.e. production facilities and capacity are available, however, cost of goods is a bit higher than the oil solutions, and the development complexity the same.

Selection of compounds for long-acting injectable formulations have historically been an afterthought, so the physico-chemical properties needed for success have not been well mapped. The selection criteria depend on the final technology; for an oil based solution, a crystalline material with high solubility (> 75 mg/mL in triglycerides, low aqueous solubility at pH 7.4 < 10 µM, high lipophilicity (ideally logD > 4) and high melting point of the free form (> 150 °C) are favoured. Ironically, these are properties usually avoided by medicinal chemists when developing an oral product. The definition of the range for logD is under discussion as recent investigations have shown that there is limited correlation between this parameter and the ability to formulate in a lipid based vehicle [61]. The choice of final presentation will be governed by solubility in oil, aqueous solubility at pH 7.4 and the stability of the formulation under forced-degradation conditions. The molecules for injection can be the parent molecules, or pro-drugs synthesized with increased lipophilicity and oil solubility to fit the parameters described above. However, pro-drugs bring the complexity of requiring the preclinical safety and exposure to both the active molecule and the pro-drug to be determined. The stability of the drug product, especially as a pre-filled sterile solution is particularly critical to ensure adequate shelf-life in tropical conditions, as a cold chain requirement for an injectable TCP-4 prophylactic would not be ideal.

Ideally, the drug should be administered either sub-cutaneously or intramuscularly. The former has the advantage that it is easier to administer, and requires less training, but there is a greater constraint on the volume that can be delivered. Given that the ultimate product will target the protection of children, infants and adults, the proposed final volume is 0.5 mL for infants and children, and 2.0 mL for adults.

The needle size is provisionally suggested at 27-gauge, to minimize discomfort during the injection. Significantly larger volumes of administration are routinely given, such as the 4 mL of microcrystalline penicillin-G benzoate through a 21-gauge needle [62], which is nevertheless very painful. However, the success of this product will ultimately depend on its acceptability by communities, and so conservative values have been taken. The needle size determines the maximum viscosity of the injected material, which may restrict the uses of certain oil-based vehicles for small molecules, or the maximum protein concentration in the case of an antibody.

Alternatives to injections are implant systems, as used in long-term contraception. These have the potential advantage of controllability, with the possibility of removal should an adverse event occur. But this approach requires potent molecules that allow limited dosage: contraceptive etonogestrel implants only contain 68 mg of drug [63]. Implants have not been developed for paediatric use.

The safety of the molecules is of paramount importance, whether considering long-acting injectables or long-acting oral molecules. In the case of any safety concern, removal of an active drug from plasma would require extensive dialysis, if feasible for the molecule, and lack of access to such a procedure could be life-threatening in resource-poor settings. It is, therefore, important to consider potential long-acting injectables within three tiers of risk:

- The first tier would consist of molecules for which extensive human systemic safety data already exist. This would apply to new formulation or a prodrug of a molecule already used for prophylaxis, such as atovaquone–proguanil. Here the safety and tolerability of the combination is well understood for oral administrations of up to 60 days, enabling an adequate assessment of the risk–benefit balance, and giving a relatively straightforward, albeit cautious path to human studies. In the initial single ascending dose studies in humans, the increase in dose should produce an increase in C_max_ and an increase in the duration of significant plasma concentration. As such, the initial first-in-human study can be de-risked by the selection of an appropriately low starting dose (this group of candidate molecules could also include new monoclonal antibodies, provided that they could be shown to be free of cross-reaction with any host targets and lack target-based enhancement of infectivity or inappropriate immune activation).
- The second tier would be prodrugs of new chemical entities with oral formulation for which initial human safety data can be collected using the oral route, allowing dosing to be halted in case of adverse events of concern. Testing of parenteral formulations could follow, but there would be no understanding of rare serious adverse events until after Phase III trials.
- The third and most difficult tier would be molecules for which no oral formulation of the active parent is possible due the physico-chemical properties of the agent. Given the required long plasma residence time, the starting dose in initial clinical trials would have to be extremely low, and the dose escalation between cohorts extremely conservative.

In some of the uses described above, the population at risk from malaria will include a considerable number of women of childbearing potential, whose pregnancy status may not be determined. For new molecules coming forward for prophylaxis, early analysis of the developmental and reproductive toxicology (DART) would be an essential part of the development strategy. Absence of any signals in such preclinical studies would facilitate the inclusion of women of childbearing age in clinical studies. This could facilitate early use of such an agent in the larger population. As discussed below, monoclonal antibodies offer a considerable advantage, since the materno–fetal transfer of IgG during the first trimester is minimal [64, 65].

The preclinical candidate will be a single molecule. There is still debate within the community as to whether the final product should be a single molecule or a combination. The potential for the selection of resistance is much lower than when a molecule is used for the treatment of uncomplicated malaria, since the molecule encounters far fewer parasites [66]. However, asymptomatic infections with quite high parasitaemias of > 5000/µL are frequently observed, and it is possible that there are pre-existing mutations within the sporozoite population, emphasizing the need for caution [67]. Resistance generation against antibodies is not expected to be a significant problem, provided that the antibodies are generated against conserved non-variant antigens from non-dividing forms, such as sporozoites, unless of course, pre-existing mutations are prevalent.

In this review a conservative approach of taking two active molecules has been adopted. This is important in early development discussions, as early human volunteer studies require the availability of formulations for both molecules that are compatible. The additional twist in this compatibility discussion is that the drug exposure profile of each molecule in the blood needs to be compatible and since this is modulated by the kinetics of release from the initial depot it may be difficult to do this for two individual molecules with different physicochemical properties. Matching pharmacokinetics is important both for duration of efficacy and for mitigating the risk of emergence of resistance during the extended tail of exposure. In theory, separate injections of the two drugs is possible, although this would be a large disadvantage. Seriously mismatched pharmacokinetics would limit the use to applications where subjects are outside of high transmission zones during the tail exposure period; again, this is possible, but not ideal. As such, any preclinical safety and toxicology studies may have to be repeated with the final formulation.

Initial proof of concept in man can be performed in volunteer infection studies with sporozoites. Recent studies with oral small molecule inhibitors of parasite liver stages have demonstrated clinically useful activity with relatively small cohorts, either using direct injections of sporozoites or insect-delivered sporozoites [26, 27]. For small molecules targeting hepatic schizonts, both approaches give similar results, with the direct injection of sporozoites being operationally easier. For antibody therapeutics targeting sporozoite antigens prior to infection of the liver, there is still a need to confirm the best approach.

The TCP-4, for a single molecule suitable for development as part of a new injectable, combination regimen for prophylaxis against malaria is detailed in Table 3.

**Table 3:** TCP-4 as part of a prophylactic combination

General considerations	Minimum essential	Ideal
Dosing regimen; adult/paediatric dose	Injectable: subcutaneous or intra-muscular, once per month, with injection volumes < 0.25 mL per molecule for infants and < 1 mL for adults via a 27 gauge or smaller needle	Injectable: subcutaneous or intramuscular once per 3 months
Pre-clinical activity	Proven liver schizont stage activity and 100% protective efficacy achieved in vivo, defined as no asexual parasitaemia after 30 days	Proven liver schizont stage activity and 100% protective efficacy achieved in vivo defined as no asexual parasitaemia after 30 days
Susceptibility to loss of efficacy due to acquired resistance	Resistance frequency in culture with erythrocytes < 10^−5^. Marker identified and no pre-existing resistance determined in the global parasite population	Resistance frequency in culture with erythrocytes < 10^−9^
Clinical protection from infection	> 80% protective efficacy (positive parasitaemia) predicted from volunteer infection studies	> 95% protective efficacy (positive parasitaemia) predicted from volunteer infection studies
Drug–drug interactions	No unmanageable risks	No interactions with other antimalarial, anti-retroviral or tuberculosis medicines or oral contraception
Safety and tolerability	Therapeutic ratio > tenfold between therapeutic exposure and NOAEL in preclinical studies and easily monitorable adverse event or biomarker for human studies. No unacceptable adverse events associated with pain, irritation or inflammation at injection site	Therapeutic ratio > 50-fold between therapeutic exposure and NOAEL in preclinical studies if not monitorable adverse event or biomarker for human studies. No adverse events associated with pain, irritation or inflammation at injection site
Preclinical DART profile	No signals in EFD and juvenile toxicology studies precluding use in children 6 months old and during 2nd and 3rd trimester pregnancies	No signals in EFD and juvenile toxicology studies precluding use in infants and women with unknown pregnancy status
G6PD deficiency status	Therapeutic dose shows minimal change in haemoglobin concentration in subjects with reduced G6PD activity. New candidate drugs show no enhanced haemolytic risk in preclinical model	Measured—no enhanced risk in subjects with reduced G6PD activity
Injectable formulation	Solutions: soluble in targeted volume based on total dose in clinically acceptable oils Suspensions: particle size controlled to give required compound release profile supporting monthly injection	Idem. Ideal formulation should be delivered in a prefilled injection device for once-in-3-months injection
Cost of single administration	Molecules consistent with a final product cost of < 5 USD per injection	Molecules consistent with a final product cost of ≤ USD 1 for infants, USD 2 for children, USD 4 for adults
Projected stability of final product under zone IVb conditions (30 °C, 75% relative humidity)	≥ 2 years	≥ 3 years

*EFD* embryo fetal development, *NOAEL* no-observed-adverse-effect-level

## Monoclonal antibodies for long-acting malaria prophylaxis

An alternative approach to protecting vulnerable populations with an injectable small molecule would be the use of a monoclonal antibody [68]. Even though malaria infection in humans does not lead to a sterilizing immune response, it is still possible that monoclonal antibodies with sufficient affinity and prolonged plasma residence times could provide protection from infection [68]. The exquisite selectivity of monoclonal antibodies means there should be no interaction with host targets, and the off-target safety issues can be avoided. Most published work is on antibodies that prevent sporozoite invasion of hepatocytes or merozoites infection of erythrocytes. These antibodies could be compared with small molecules for TCP-4 (hepatic schizonts) or TCP-1 (erythrocytic schizonts) with the subtlety that the antibodies prevent the initial infection of the respective cell types, whereas small molecules prevent the replication of the parasite within the host cells. In both cases the time window for action of an antibody is a critical element which distinguishes the antibody profile from the drug profile. An antibody against sporozoites must be able to act in the short time that it takes for sporozoites to move from the skin to the liver, typically within 30 min. Antibodies against merozoites must exert their effect in the short window when the merozoites are present in free circulation, typically 30 s.

Given the high commercial price of many existing antibody therapies, it is often assumed that the cost of antibody therapy would be prohibitive in most malaria-endemic countries. Provided antibodies are expressed at high levels (4 g/L), and are stable, hence the production costs can drop from around $100–300/g [69] to estimates of around $35–85/g [70], with room for a further decrease in costs, projected to be $20/g at the multi-tonne scale.

As with small molecules, an intramuscular or sub-cutaneous presentation is probably preferable to intravenous injection, and so a limiting factor is the volume of administration. The amount of antibody is, therefore, limited by the achievable concentration. Although concentrations as high as 200 mg/mL have been reported for subcutaneous injection, a more conservative target is 100 mg/mL [71]. Based on a 0.5 mL injection volume for children and 2 mL for adults, this sets a dose ceiling of 50 mg and 200 mg, respectively (Table 2). This in turn sets an optimized cost maximum for the antibody at $1.75 for children and $7 for adults, excluding the costs of vials and distribution. These price estimations are on the borderline of what might be acceptable, in comparison with the pricing for a malaria vaccine. Any decrease in dose, frequency of administration or cost of manufacturing would further bring down these costs.

To achieve long-term prophylaxis, mutations in the Fc region are needed. Monoclonal antibodies tend to be IgGs, with plasma half-lives of 20–25 days [72, 73]. This half-life is partly controlled by FcRn receptor-mediated elimination, and also depends on the antigen abundance. Mutating the IgG Fc region to increase the interaction with its receptor FcRn, at the acidic pH encountered in the lysosome, prevents elimination of the antibody in the lysosome, favouring its recycling. This can extend the circulation duration of an antibody threefold, as has been achieved for bevacizumab and cetuximab [74], through simple Met428Leu and Asn434Ser mutations. Other approaches to half-life extension make the antibody bulkier by fusion, mulitmerization or pegylation, but these approaches would dramatically increase the cost of goods [75].

An antibody could be compared directly with a small molecule in the animal model. The goal would be an 80% protection from infection, minimally for 1 month and ideally for 3 months. Given the restrictions on injection volume, this would require administration of 2 mg/kg of each active molecule in adults and 2–5 mg/kg in children. As for small molecules, the simplest preclinical model for therapeutic antibodies would be the FRG chimeric mouse model with engrafted human hepatocytes, discussed earlier. Allometric scaling for a series of antibodies [76] suggests that systemic clearance is proportional to the body weight raised to a power of 0.91 (Cl = a·BW^b^), a much higher number than seen with small molecules. Therefore, allowing for the difference in body weights for humans and mice, this translates approximately into a twofold difference in the mg/kg dose between humans and mice, setting an efficacy threshold in mice of an 80–200 mg total dose. This could be related back to a cellular potency target EC_50_ < 100 pM (< 15 ng/mL), based on modelling of various affinity and dissociation rates [77, 78]. It is important to demonstrate in such studies, that there is no risk of stimulating antibody-dependent enhancement [79].

As mentioned above, the exquisite selectivity of antibodies means that off-target effects are minimized. Anti-sporozoite or anti-merozoite antibodies do not target host antigens. Therefore, toxicology studies should be less complicated compared to other indications. Fetal transfer in the first trimester is reported to be minimal; the required Fc receptor for the transfer of IgGs is hardly detectable in the placental syncytiotrophoblast during the first trimester [80]. Specific studies on monoclonal antibodies in pregnancy are rare, given that most antibodies are given for treatment of cancer or autoimmune diseases. Several studies have reported that multiple sclerosis patients in early stages of pregnancy exposed to natalizumab or alemtuzumab did not have an increase in the frequency of abortions [81]. In Inflammatory Bowel Disease, the European evidence-based consensus is to continue treatment with anti-TNF throughout pregnancy, and the practice appears safe [82]. This means that antibody prophylaxis could potentially be given to young women whose pregnancy status is unknown, which is a tremendous advantage over new chemical entities [64].

Antibody discovery tends to be much faster than for small molecules, with a much higher probability that an individual project will deliver a development candidate [83]. However, once a development candidate is defined, the production of high-grade material for the initial clinical trials is much more expensive. Typical costs are in the region of $5 million, which contrasts starkly with around $100 k for a small molecule. This combination of an easier pathway to candidate identification, but a more expensive decision stage gate, means that it is very important to have clear parameters for the TCP.

## Clinical and regulatory strategy of long-acting malaria prophylaxis

For new chemical entities and antibodies the Phase I single ascending dose study in healthy volunteers would aim to achieve the plasma concentration predicted from studies using the FRG-mice [84]. At this concentration, prophylactic activity could be assessed in human volunteer malaria infection studies [26, 27]. For new chemical entities, the safety and efficacy in human volunteers should ideally be established initially using an oral formulation, which will help guide the safety recommendations for dose escalation of the injectable in Phase I.

**Fig. 1:**
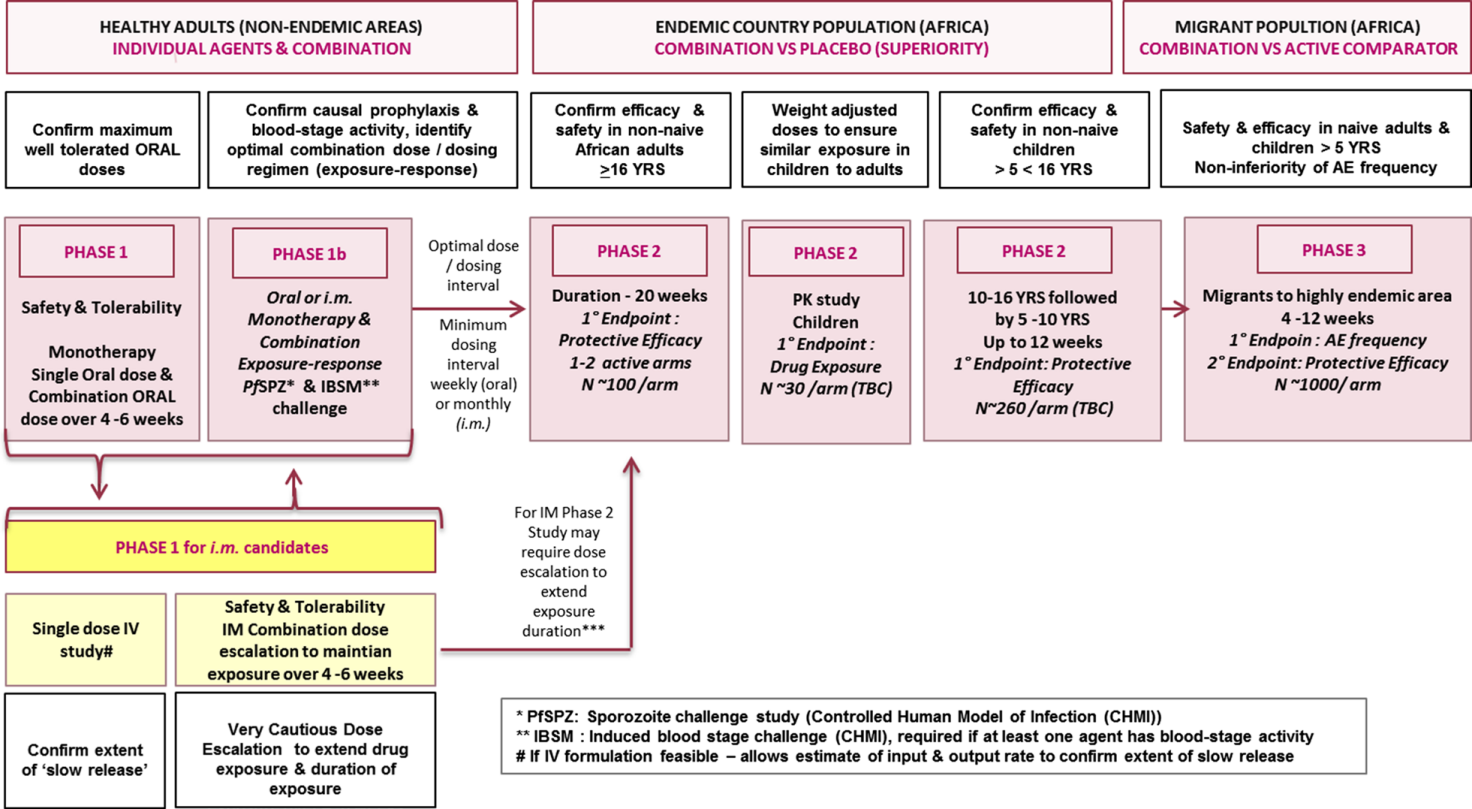
Proposed high level clinical development plan for evaluation of a long acting malaria prophylactic

In parallel, it is important to investigate the potential combinations of two molecules that can be brought forward. Simulations using data from preclinical cytochrome P450 and transporter assays can help eliminate combinations with likely problematic drug–drug interactions. Additivity and possible synergy between two molecules can be studied in the FRG SCID mouse. Exposure–response analyses of these studies also allow modelling of the doses proposed for Phase II trials. In addition, the modelling has the potential to demonstrate the contribution of the individual compounds to overall efficacy, addressing the ‘combination rule’ required by the US FDA. The safety of combinations needs to be investigated in healthy adult volunteers combination studies before moving to Phase II trials in infected but asymptomatic subjects. A flow chart for the selection and optimization of new molecules with potential chemoprotective activity is shown in Fig. 1.

The Phase II and Phase III clinical strategy is based on the lessons learned from the development of atovaquone–proguanil [55, 85, 86] for malaria prophylaxis. Atovaquone–proguanil was first developed as a malaria treatment, and was subsequently shown to be efficacious in prophylaxis, using a daily dosing regimen. However, in the case of a new injectable protective regimen, the goal is to try to achieve registration for a prophylaxis indication *directly*. Such a development plan for prophylaxis was presented for initial discussion with regulators (the UK Medicines and Healthcare products Regulatory Agency, MHRA) in the context of an oral drug combination therapy. Similar considerations would apply for antibodies. Two main clinical trial designs are proposed to support registration for a prophylaxis indication (Fig. 1). Phase II studies would be carried out in subjects resident within an endemic area, and Phase III studies would be undertaken in a genuine migrant population, such as seasonal workers, or boarding school children. Studies would be conducted in highly endemic areas, to obtain sufficient discriminatory power versus comparator to demonstrate efficacy. Since it is thought that relatively few children less than 5 years of age are likely to be travelling from malaria-free to malaria endemic areas, recruitment into clinical trials of this younger population may be problematic. For this reason, the plan would be to seek approval for subjects over 5 years in the first instance. Recruitment of younger children is likely to be easier following approval of the medicine for prophylaxis in the older population, when sufficient evidence of safety and efficacy is available.

To support a stringent regulatory authority registration for prophylaxis, two Phase II studies are proposed in a non-naïve population (Fig. 1), the first carried out over 20 weeks in adults, and the second carried out over 12 weeks in children of 5 years and above. Both studies would be randomized, double-blind placebo-controlled studies to evaluate the prophylactic efficacy, safety and tolerability in non-naïve subjects in regions of Africa with a high incidence of malaria. The primary endpoint would be protective efficacy (PE). A key aspect of this design is that a sterilizing cure is administered to study subjects to clear both blood-stage parasites and gametocytes prior to assessing the prophylactic efficacy of the drugs under investigation. This design is, therefore, a useful model of uninfected populations travelling to endemic areas, although the subjects will have the benefit of partial-immunity from previous infections. From a study design perspective, this approach has the significant advantage of allowing the use of a placebo comparator, thus providing a direct measure of PE (defined as: 1-failure rate active treatment/failure rate of placebo).

In the first of these two studies, two to three combination dose levels or regimens could be evaluated, using an exposure–response approach in a relatively small number of subjects, to identify the optimal dose and frequency of dosing. Following the identification of the dosing regimen that provides the target protective efficacy with a practical dosing frequency, and with acceptable safety and tolerability, a small pharmacokinetic study or Phase II run-in could confirm that the dose adjustments in children achieve the target drug exposures.

This would then be followed by a Phase II study in children which would test the selected regimen in non-naïve school children resident in an endemic area. The design would involve a step-wise, age de-escalation approach. A full safety review would be carried out before enrolling each new cohort, descending via cohorts of approximately 30 10–16 year olds, to 5–10 year olds. The decision to move to Phase III would be based on the likelihood of achieving protection over 3 months. Such protection would ideally be in a population which had no protection supplied by immune memory. The demonstration of activity only in a population with some background immunity could still be useful—for example providing protection in pregnancy, or as a potential replacement for SMC. Since the effect of partial immunity on the efficacy in the Phase II population cannot be estimated directly, a somewhat higher efficacy than required for Phase III could be set as the threshold to move from Phase II to Phase III. The acceptable efficacy for the prophylaxis indication will ultimately depend on the likely use cases and validation in further discussion with the wider community, balancing efficacy and aspects such as deployment practicality, efficacy and cost per Disability Adjusted Life Year (DALY).

The use of a placebo in adults and children in the Phase II studies in areas of high malaria incidence was strongly recommended in initial discussions with regulatory authorities because this is the only circumstance in which the true attack rate is known, and hence the true protective efficacy can be measured. The population enrolled in this study would ordinarily be exposed to sub-clinical malaria infection which would otherwise remain untreated. All subjects would receive an initial sterilizing cure for this sub-clinical malaria, and benefit from close clinical safety monitoring during the study, as well as standard-of-care treatment upon detection of positive parasitaemia during study conduct. The acceptability of placebo controlled trials in adults and in children will need additional discussion with the Ethics Committees at individual sites, to take account of local concerns on this topic. There are sometimes ethical objections to placebo injections, especially if repeated blood sampling was undertaken. An alternative would be to combine the placebo or experimental drug with a proven vaccine against a different disease.

The protective efficacy, safety and tolerability would then be confirmed in a Phase III study in the target population. These are adults and children older than 5 years travelling from geographical areas with no, or a very low risk of malaria infection, to geographical areas of significant risk of malaria infection. Ethically, these studies need to be active controlled studies; oral atovaquone–proguanil being the most likely comparator. The protective efficacy of both atovaquone–proguanil and the NCE (new chemical entity) is expected to be high, therefore a very low rate of malaria infection (*‘attack rate’*) would be expected in this population, meaning that very large numbers of subjects would be required to demonstrate non-inferiority of the new medicine. For this reason, in the development of atovaquone–proguanil, the primary endpoint in Phase III studies was the overall frequency of any adverse events assessed at 7 days after leaving the endemic country, with protective efficacy as a secondary endpoint. In these comparator studies, confirmed *P. falciparum* malaria occurred in 0/486 and 0/477 subjects receiving atovaquone–proguanil and mefloquine, respectively [55], and in 0/501 and 3/507 subjects receiving atovaquone–proguanil and chloroquine–proguanil, respectively [87], illustrating the difficulty of this approach. Although atovaquone–proguanil is the best comparator, such studies have the additional complication of a lack of marketing authorization in many of the areas ultimately targeted.

Demonstrating non-inferiority of protective efficacy would as mentioned above require very large trials. Making a number of assumptions about the risk of developing malaria for travellers from Europe, Canada and South Africa to East Africa who do not take prophylaxis, the average duration of travel and the efficacy of a prophylactic treatment, Høgh et al. [87] estimated that assuming a protective efficacy of chloroquine–proguanil of 72%, a study in travellers designed to show that a new anti-malarial drug with 95% efficacy is better than chloroquine–proguanil, assuming a 80% power and a 5% significance level, would require more than 16,000 participants While a number of the assumptions made would not necessarily apply to African migrants traveling for work and for schooling, perhaps for longer periods, and exposed to higher bite rates (due to greater endemicity, and a less protective living environment), nevertheless unrealistically large numbers may be required to test for non-inferiority of efficacy. Indeed, given the reported 98.5–100% efficacy for atovaquone–proguanil, demonstration of non-inferiority would not be expected, but demonstration of high efficacy of the NCE would be required. Therefore, similarly to the development of atovaquone–proguanil, for a long-acting injectable prophylactic trial in adults proposed here (Fig. 1), the primary endpoint would be non-inferior safety and tolerability compared to atovaquone–proguanil, with prophylactic efficacy as a secondary endpoint. Hence the required sample size of any future Phase III studies will be based on the most frequent and clinically relevant adverse event(s) of both test medicine and comparator, and the non-inferiority margin will be set based on a proposed clinically relevant difference in adverse event rate.

In migrant populations a number of potential approaches have been published, including enrolment of subjects in Travel Clinics [55, 87], protection of soldiers on active duty, boarding school children or groups of workers deployed for short periods in hyper endemic regions [88].

In initial discussion with MHRA, a total safety population of approximately 3000 subjects exposed to the clinical dose was deemed a sufficient safety data package for registration of an oral prophylaxis, provided no significant safety signals are detected. The breakdown numbers of adults *versus* children was not considered critical as long as there is a good spread of age across the study.

## Conclusions

Several factors have driven a renewed evaluation of the role of prophylaxis in the malaria elimination agenda over the last few years:

- First, the general acceptance that countries that are undergoing elimination will have internal populations migrating from low transmission zones to those of high transmission, for example going from the south to the north of Zambia. In the past, the prophylaxis of migrant populations was considered only to be commercially relevant for protecting western tourists and soldiers. However, it is now clear that cost-effective solutions must be found to protect migratory populations in low- and middle-income countries.
- Second, in countries that have eliminated malaria and are ‘maintaining zero’ [12], there is the risk of re-introduction and epidemics, and in the absence of a fully effective vaccine, an alternative approach to protection is needed for populations at risk of an epidemic. A special subcategory is the need for malarial prophylaxis during fever outbreaks such as the Ebola crisis in West Africa, to reduce the risk of malaria at the height of the epidemic and to protect health care workers.
- Third, there have been tremendous successes in the last 5 years using SMC, which involves giving a full treatment course of anti-malarials every month. A regimen which was truly optimized for prophylaxis, and available as an injection would have potentially major benefit. This latter point has come into focus recently because of the stagnation in the reduction of malaria incidence globally [44]. One of the strategic responses here is to ask what else can now be done in high-burden countries to achieve significant reductions in morbidity, and new prophylaxis regimens that were simpler to administer than current SMC could have an even bigger impact and which could potentially be given in areas with perennial high transmission.

Several factors lead to some optimism for the future:

- First is the availability of new chemotypes with activity against the malaria parasite, which opens up the possibility of focusing on new medicines specifically designed for prophylaxis. This is an appealing situation since, ideally, the same medicine should not be used for both the protection and the treatment of malaria within the same geographic locale. To date, none of the new molecules has a half-life equivalent to that seen from oral doses of 4-aminoquinolines or the 8-aminoquinoline tafenoquine, hence the need for slow-release injectable formulations, to provide the appropriate duration of cover.
- Second, the availability of better in vitro *P. falciparum* liver stage assays and animal models, including a murine model of the hepatic infection of *P. falciparum* allows the comparison of different molecular classes in vivo. Standardization of protocols for such assays will be an important driving factor over the coming years, allowing head-to-head comparisons of small molecules and monoclonal antibodies.
- Third, in experimental medicine, the arrival of robust supplies of GMP-standardized sporozoites has enabled testing of new chemical series in human volunteer infection studies. These data, or alternatively those obtained in insect-driven infection studies, allow early identification of compounds with activity in humans, and estimation of human effective doses for full-scale clinical studies.
- Finally, new developments in formulation technologies allow the development of some of the molecules with prophylactic activity as long-acting injectables. This can be achieved by formulation of the parent compound or development and formulation of pro-drugs. Developments in HIV, oral contraception and antipsychotic medicines have shown that slow release allowing protection for one or even several months is possible, with acceptable dosing volumes and needle sizes. These studies also underline the need for highly potent molecules, to minimize cost, and to maximize acceptability.

Taken together, there are many reasons to be optimistic about the probability of identifying and developing long-acting injectable formulations. What is important is that a common language and common standards are applied to assess the different candidates, and on this basis Target Candidate Profiles are proposed. In addition, the route to registration will be a new one, not necessarily proceeding via the approval of medicines for a treatment indication. As such, new target product profiles and the use cases supporting their delivery in the field are important. This document contains proposals for such profiles, in the full knowledge that over the next few years clinical data will become available providing many lessons that aid in further refinement of the next generation of profiles. More discussion is needed with experts in the field and the bigger malaria community e.g. on the clinically relevant level of efficacy to be targeted. Furthermore, MMV is preparing clinical development strategies for new prophylactic medicines and engaging stringent regulatory authorities in early discussions.

## Abbreviations

ACT: artemisinin combination therapy
CHMP: Committee for Medicinal Products for Human Use
CSP-1: Circumsporozoite Antigen-1
DADDS: 4-4′-diacetylaminosulphone
DALY: Disability Adjusted Life Year
DART: developmental and reproductive toxicology
DHFR: dihydrofolate reductase
DHODH: dihydroorotate dehydrogenase
DHPS: dihydropteroate synthase
EF2: Elongation Factor 2
EFD: embryo and fetal development
EMA: European Medicines Agency
FDA: Food and Drug Administration
FRG mice: mice that lack functional Fah, Rag-2 and Interleukin 2 receptor common gamma chain genes
G6PD: glucose-6-phosphate dehydrogenase
GDP: Gross Domestic Product
GSK: GlaxoSmithKline
HIV: human immunodeficiency virus
MHRA: Medicines and Healthcare products Regulatory Agency
MMV: Medicines for Malaria Venture
NCE: new chemical entity
NOAEL: no-observed-adverse-effect-level
*P*.: *Plasmodium*
PE: protective efficacy
PI-4 kinase: phosphoinositol-4 kinase
PrEP: pre-exposure prophylaxis
(S)AE: (severe) adverse event
SCID: severe combined immunodeficiency
SMC: seasonal malaria chemoprevention
SP–AQ: sulfadoxine–pyrimethamine plus amodiaquine
TCP: target candidate profile
TPP: target product profile
WHO: World Health Organization
